# Fatigue Crack Growth Behavior of the MIG Welded Joint of 06Cr19Ni10 Stainless Steel

**DOI:** 10.3390/ma11081336

**Published:** 2018-08-02

**Authors:** Lanqing Tang, Caifu Qian, Ayhan Ince, Jing Zheng, Huifang Li, Zhichao Han

**Affiliations:** 1 Department of Chemical Mechanics Engineering, Beijing University of Chemical Technology, Beijing 100029, China; Lanqingtang@mail.buct.edu.cn (L.T.); lihf@mail.buct.edu.cn (H.L.); hanzhichaohzc@163.com (Z.H.); 2 Purdue Polytechnic Institute, Purdue University, West Lafayette, IN 47906, USA; zheng338@purdue.edu; 3 Department of Mechanical, Industrial & Aerospace Engineering, Concordia University, Montreal, QC H3G 1M8, Canada

**Keywords:** fatigue crack growth, MIG welded joint, microstructure, residual stress, 06Cr19Ni10

## Abstract

In this paper, the fatigue crack growth behavior of the base metal (BM), the weld metal (WM) and the heat-affected zone (HAZ) in the metal-inert gas (MIG) welded joints of the 06Cr19Ni10 stainless steel are analyzed and studied. Results of the fatigue crack propagation tests show that a new fatigue crack initiates at the crack tip of a pre-existing crack, then propagates perpendicular to the direction of cyclic fatigue loads. This observation indicates that the original mixed-mode crack transforms into the mode I crack. The WM specimen has the largest fatigue crack growth rate, followed by the HAZ specimen and the BM specimen. To illustrate the differences in fatigue crack growth behavior of the three different types of specimens, metallographic structure, fracture morphology and residual stresses of the BM, HAZ and WM are investigated and discussed. The metallographic observations indicate that the mean grain size of the HAZ is relatively larger than that of the BM. The fractographic analysis shows that the WM has the largest fatigue striation width, followed by the HAZ and the BM. It is also found that the depth of dimple in the WM is relatively shallower than the one in the HAZ and BM, implying the poor plasticity behavior of the material. Analysis results of the residual stress analysis demonstrate a high level of tensile residual stress appearance in the WM and HAZ.

## 1. Introduction

06Cr19Ni10 steel belongs to the family of Cr-Ni austenite stainless steels, which is of particular interest to a wide range of fields such as petrochemical, electricity, metallurgy, aircraft and pressure vessel industries due to its excellent corrosion resistance, high heat resistance and prominent mechanical properties [1]. Generally, conventional arc welding techniques such as gas welding, arc welding, pressure welding and braze welding are considered as the most convenient and time-efficient methods to join metal/alloy parts and produce high-quality weld joints with several unique advantages [2,3]. In particular, as an important member of the arc welding family, metal inert gas (MIG) welding is thought to be a suitable method for austenite steel joining due to the ease of operation and relatively high productivity. In the welding process of the MIG, both the arc and the weld are protected by a gas shield [4]. However, rapid thermal cycles cause metallic phase transformation during the welding process and ultimately lead to the inhomogeneity of the microstructure and the material composition of welded structures. Hence, a typical weld joint is classified into three basic zones, namely, the base metal (BM) zone, the weld metal (WM) zone and the heat affected zone (HAZ) on the basis of the material characteristic and the metallographic structure [5,6].

Metallurgical defects like pores, voids, inclusions and cracks, stress concentration and tensile residual stresses are inevitably introduced in the WM and the HAZ during the welding process, which deteriorates both mechanical and fatigue properties of weld joints. Accordingly, when the weakened weld joint is subjected to a fluctuation of the thermal and mechanical cyclic loads in real service conditions, fatigue cracks are prone to be induced in the weld joint. Thus, the weld joint, especially for the WM and HAZ, is regarded as a potential weak link of welded structures from the perspective of fatigue damage [7,8]. In the past decades, numerous researchers have evaluated fatigue behaviors of different metals and alloys but few of them focused on investigating fatigue properties of different areas in weldments [9,10,11,12,13,14]. Parkers et al. [9] studied fatigue properties of a DP980 weld joint. The authors found that the fatigue strength of the weld joint was relatively lower than that of the BM. It was also reported that high cycle fatigue failure occurred in the BM for high-stress amplitudes, and in the WM and the HAZ for low-stress levels. Qiao et al. [10] evaluated fatigue crack propagation characteristics in the BM, HAZ and WM of an A7N01 weld joint. Their results demonstrated that a fatigue crack initiated near the WM in comparison to the BM and the HAZ, and that the WM had the largest crack growth rate (CGR). Tagawa et al. [11] evaluated fatigue properties of cast aluminium weld joint. It was found that fatigue times of MIG weld specimens were relatively shorter than those of base metal specimens. Basak et al. [12] researched fatigue behavior of MIG-welded DP600 steel joint. They found that, for the near-threshold values of Δ*K*, the crack growth rate of welded joints was higher than that of the base metal specimen. Gaur et al. [13] investigated the fatigue behavior of MIG-welded Al-5083 and Al-5183 alloys at different stress-ratios. The results showed that the fatigue limits and lives of both materials did not differ significantly, but they decreased with the increasing *R*-ratios.

There are numerous influential factors that affect fatigue behavior of weld joints, and one of them is regarded as the material microstructure. The effect of microstructures is considered to be of great significance because of the metallurgical stress concentration and the presence of the microstructural defects [15,16]. Chan et al. [15] summarized the influence of microstructures on the crack initiation for different materials. The authors found that a microstructure parameter such as the grain size can influence fatigue limits by introducing internal textural stresses. Cao et al. [16] studied microstructure and fatigue properties of the 304H austenite steel. It was indicated that the coarse martensitic structure in the WM tended to produce weaker fatigue strength of the weld joint.

Apart from the material microstructure, it is widely reported that residual stresses also have a non-negligible influence on the fatigue behavior of welded structures [17,18,19,20]. In recent years, fatigue failures of welded structures such as bucket wheels, railway rails and lifting platforms have been noticeably arising [21,22,23]. Basically, tensile residual stresses are known to have detrimental effects on decreasing fatigue life of welded structures [24,25]. Ngoula et al. [12] evaluated the influence of residual stresses on both the effective cyclic J-internal and fatigue life of weld joints. Their results showed that compressive residual stresses were found favorably to increase fatigue lives of weld joints and the effect of welding residual stresses would not be neglected in the majority of cases. Teng et al. [13] adopted a finite element (FE) method to investigate the influence of welding residual stresses on the fatigue life. They found that the reduction in tensile residual stresses improved the fatigue performances of A36 weld joints.

Although many studies related to studying the fatigue behavior of weld joints have been conducted, yet there are still numerous questions remain to be clearly answered. The purpose of this work is to evaluate fatigue crack growth behavior of the BM, the WM and the HAZ specimens. Furthermore, metallographic structures, fracture morphology and residual stresses are also investigated and compared to make comprehensive assessments of the difference in fatigue crack growth behavior of the three different zones.

## 2. Experimental Procedures

### 2.1. Specimen Preparation

**Figure 1:**
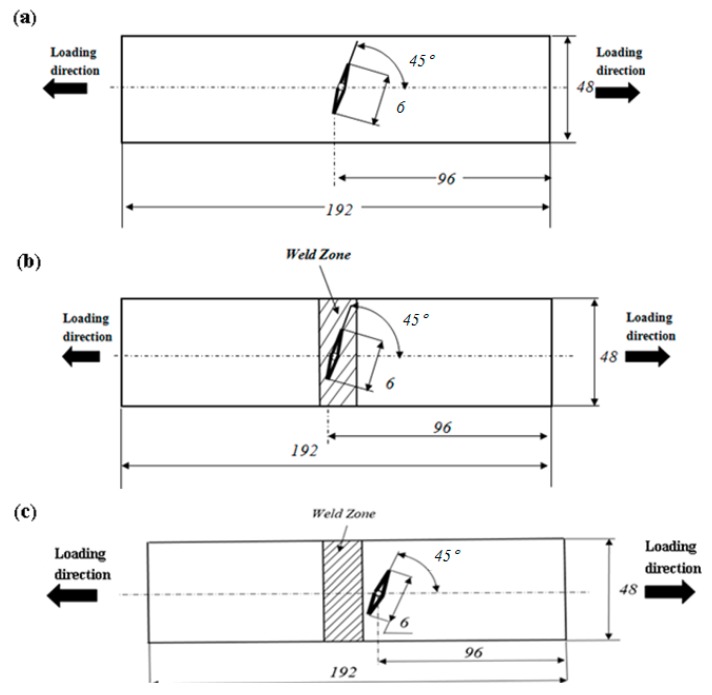
Three types of center-cracked fatigue specimen (mm): (**a**) the BM specimen; (**b**) the WM specimen; (**c**) the HAZ specimen.

Test specimens of the BM were fabricated from hot rolled 06Cr19Ni10 steel plates in the material rolling direction. The investigated weld joints were constructed by the metal-inert gas (MIG) welding method using an ER308L electrode (Baiyi Industry Co., Changzhou, China). Table 1 and Table 2 summarized the chemical compositions of the 06Cr19Ni10 and the ER308L and the welding parameters of the MIG welding process, respectively. After welding, dry plama cutting was adopted to produce smooth-faced fatigue specimens with the size of 98 mm (length) × 48 mm (width) × 6 mm (thick), as shown in Figure 1. Specially, the air compressor (Peak Scientific Co., Manchester, UK) was used to control the temperature of specimen, thus preventing altering the residual stress state. Then, an inclined crack with an angle β of 45° and a length of 6 mm was introduced in the center of each fatigue specimen by the electric discharge machining (EDM, DM300K, HANQI Co., Suzhou, China). Finally, to observe the crack initiation process easily, the specimen surface was polished to be a mirror surface. Here, all the experimental specimens were classified into three different types on the basis of the crack’s position: the BM specimen, the HAZ specimen and the WM specimen.

### 2.2. The Fatigue Testing and Microstructure Observation

Fatigue crack growth (FCG) tests were performed using a 100 kN INSTRON 8800 servo fatigue machine (Instron company, Boston, MA, USA) at room temperature. A Scalar DG-3 digital camera (New Xinhua Co., Beijing, China) was employed to obtain a real-time observation and measurement of fatigue crack lengths on the specimen surface. The values of maximum cyclic stress, the stress ratio and the loading frequency used in FCG tests were 200 MPa, 0.1 and 15 Hz, respectively. As for the intermediate Δ*K* regime, the Paris-Erdogan law was adopted to describe the relationship between fatigue crack growth (FCG) rate, *da*/*dN* and the stress intensity factor (SIF) range, Δ*K* [26], as expressed in Equation (1).

(1) $$\frac{da}{dN}=C{\left(\Delta K\right)}^{m}$$

where the coefficient *C* and the exponent m are material-dependent quantities obtained from curve-fitting of Equation (1) with experimental FCG data. The microstructures and fracture surfaces of the BM, the HAZ and the WM specimens were also investigated by using an optical microscope and a scanning electron microscopy (SEM, SU3500, Hitachi Co., Tokyo, Japan), respectively.

### 2.3. Simulation and Measurement of Welding Residual Stresses 

#### Residual Stress Simulation

**Figure 2:**
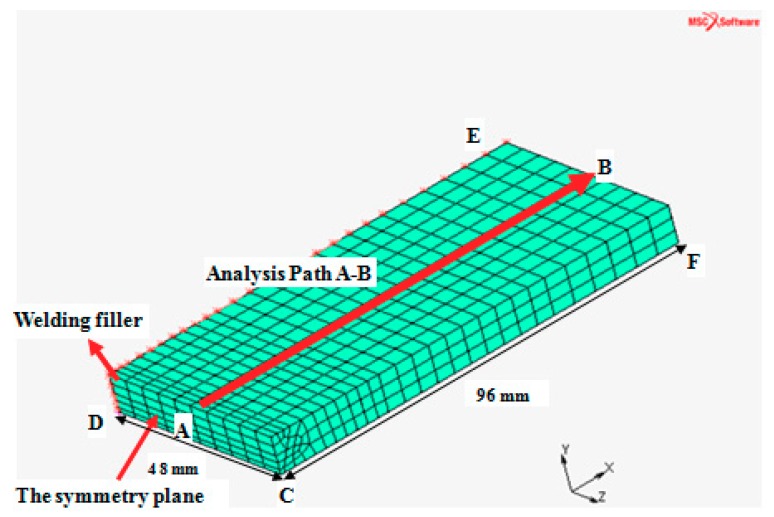
FE model of the MIG butt welded joint.

The thermo-mechanical analysis of the MIG welding of the 06Cr19Ni10 stainless steel was performed to simulate welding process. As shown in Figure 2, only one half of the actual test specimen was modeled with 3D solid elements owing to the overall symmetry boundary condition. The commercial MSC-Marc software package (MSC Marc 2016, MSC Software Corporation, Los Angles, CA, USA) was employed to construct a three-dimensional finite element (FE) model of the butt weld joint. The total numbers of nodes and elements in the model were set to be 9054 and 2108, respectively. It can be seen in Figure 2, different element sizes for the mesh were used. Specially, coarse FE meshes were generated in the base metal to enhance the efficiency of calculation. While refined FE meshes were created in the weld region to achieve accurate modeling results. Welding analyses were considered to be sensitive to the element size. Therefore, the convergence study was carried out to obtain the element size of the FE model. First, the elements of the welding FE model were refined with the proportion of 2:1. Then, multiple FE computations before and after element refinement were performed. If there was a small difference (≤5%) in FE results between the original FE model and the refined FE model, the FE solutions were considered to have a good convergence. Finally, the minimum element size in this study was chosen to be 1 mm × 1 mm × 5 mm. The A–B path in Figure 2 was selected to evaluate the residual stress distribution in the WM, the HAZ and the BM of the butt weld joint. Static boundary conditions were imposed on the FE model to represent clamping conditions during welding of test specimens. Due to the symmetry of the FE model, the constraint of *x* directional displacement was applied at the symmetrical plane. Besides, the *y*-displacements of C, D, and E on the bottom surface were constrained; and the point F was fixed in the *z*-direction, as shown in Figure 2. 

**Figure 3:**
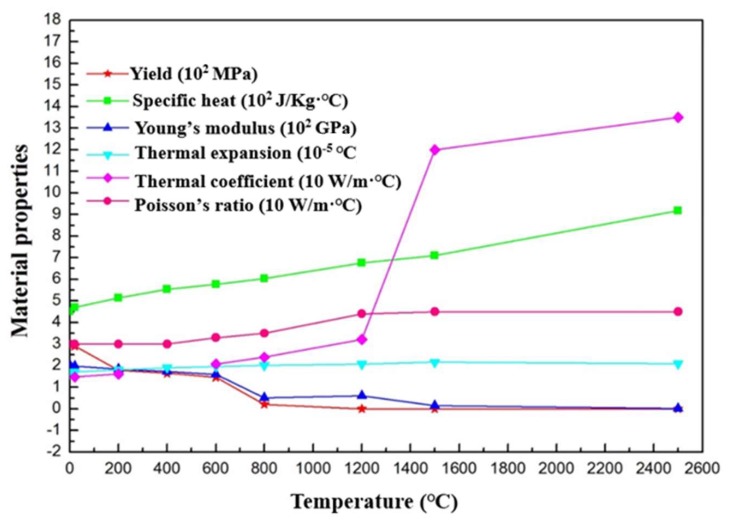
Thermal and mechanical properties of 06Cr19Ni10 steel.

As for thermal boundary conditions, the radiation and convection heat loss was taken into consideration, using a total film coefficient, 20 W/K·m^2^. The initial ambient temperature was assumed to be 18 °C. The temperature-dependent material properties such as passion’s ratio, yield stress, specific heat, Young’s modulus, thermal coefficient and thermal expansion were obtained from Deng and Murakawa’s work [27], as listed in Figure 3. In a thermal welding simulation, the material’s properties were highly nonlinear. To simulate the heat flow in the welding process, a moving heat source model was applied on the surface of weld pool. In this study, a double-ellipsoid heat source model proposed by Goldak [28] was adopted to express the heat flux in the numerical simulation of the welding process. This model consisted of two ellipsoidal heat sources with the similar geometries, as depicted in Equations (2) and (3).

The forward heat source was described as

(2) $$q_{1}(x,y,z)=\frac{6f_{f}\eta UI}{\pi abc_{1}\sqrt{\pi }}\exp(-\frac{3x^{2}}{a^{2}}-\frac{3y^{2}}{b^{2}}-\frac{3z^{2}}{c_{1}^{2}})\;$$

Similarly, the rear heat source was described as

(3) $$q_{2}(x,y,z)=\frac{6f_{r}\eta UI}{\pi abc_{2}\sqrt{\pi }}\exp(-\frac{3x^{2}}{a^{2}}-\frac{3y^{2}}{b^{2}}-\frac{3z^{2}}{c_{2}^{2}})\;$$

where UI was representing the heat input, a, b and *c* were referring to the geometric size, the heat fractions $f_{f}$ and $f_{r}$ were representing the forward fraction and the rear fraction of heat source, respectively.

Residual stress measurements were carried out by X-ray diffraction (XRD, Stresstech Corporation, Helsinki, Finland) using XSTRSSTECH G2 in order to verify the reliability and validity of simulation results. The measurements were based on sin^2^ method with angles varied from −45° to 45° [29]. The residual stresses can be determined using the following equation:

(4) $$\sigma ={\left(\frac{E}{1+\nu }\right)}_{\left(hkl\right)}\frac{1}{d_{0}}\left(\frac{\partial d_{\psi }}{\partial \sin^{2}\psi }\right)$$

where *E* is the Young’s modulus, *υ* is the Poisson ratio, *ψ* is the incident angle, *d*_0_ is the crystallographic *d*-spacing under no stress condition, *d_ψ_* is the crystallographic *d*-spacing under stress condition, *h*, *k* and *l* are crystal face exponents.

**Figure 4:**
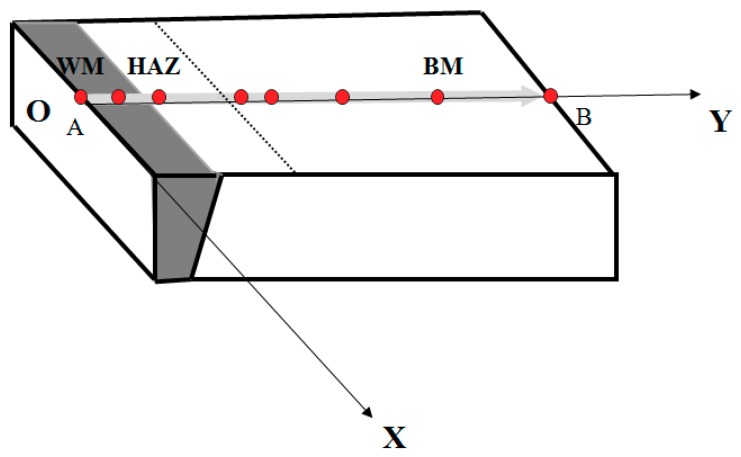
Measuring points of residual stress.

As shown in Figure 4, point A was determined as the origin of the coordinate system, path A–H and path A–B were defined as the *x*-axis and the *y*-axis, accordingly. Eight points in the *y*-axis with the coordinates of (0, 0), (0, 3), (0, 6), (0, 9), (0, 15), (0, 36), (0, 60), (0, 96) in mm length were set as the measuring points to analyze residual stresses in the BM, HAZ and WM regions of the welded joint. 

## 3. Results and Discussions

### 3.1. FCG Paths

**Figure 5:**
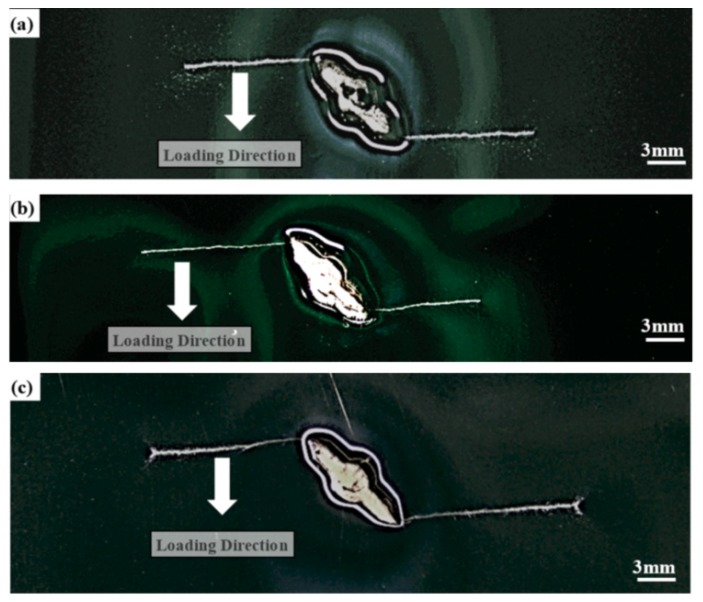
Crack propagation paths: (**a**) the BM specimen; (**b**) the HAZ specimen; and (**c**) the WM specimen.

Figure 5a–c depict the optical macrographs of crack propagation paths for the BM, the HAZ and the WM specimens, respectively. Obviously, fatigue cracks initiated at both top and bottom tips of the inclined pre-fabricated crack for all three types of specimens, and then propagated along the direction perpendicular to the direction of fatigue loadings, as illustrated in Figure 5a–c. This observation demonstrated that the original mixed-mode cracks changed into the mode I cracks. The specimen’s type and the crack location had a negligible effect on the fatigue crack growth path in weld joints. Similar phenomena had been observed in fatigue crack propagation tests of various materials [30,31]. In general, the crack advanced under the control of the primary normal stress, after the crack propagated a certain distance of over 250 μm which explained why all the cracks propagated in the direction perpendicular to the fatigue loading [32].

### 3.2. FCG Rate

**Figure 6:**
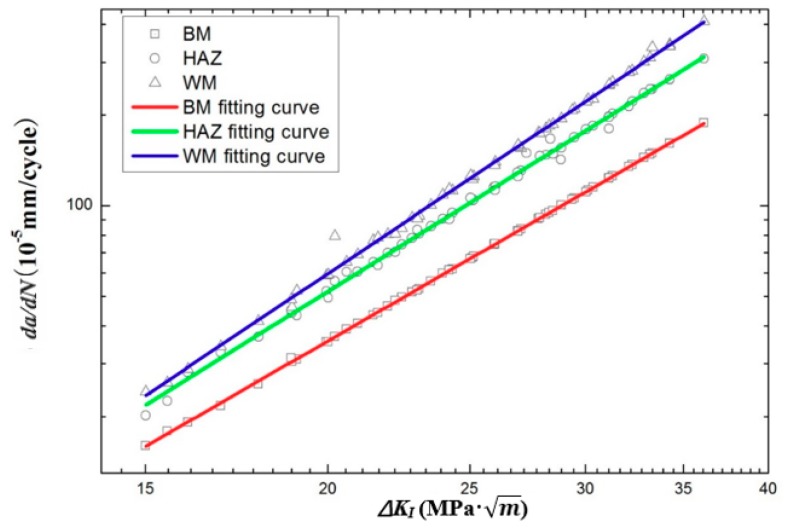
The Paris curves in BM, HAZ and WM specimens.

The fatigue test data were dealt with the seven point incremental polynomial method and the finite element analysis (FEA) to obtain the FCG rate, *da*/*dN* and the SIF range, Δ*K*. The linear regress analysis between *da*/*dN* and Δ*K* were performed with the Origin software package (Origin 9.0, Origin lab Corporation, Northampton, MA, USA). In the Origin software, the log-log coordinate was chosen to describe the parameter relationship. Δ*K* and *da*/*dN* were inputted as the horizontal and vertical coordinates, respectively. Figure 6 plots fatigue crack propagation curves of the FCG rate, *da*/*dN* versus the SIF range, Δ*K* for the BM, the HAZ and the WM specimens at the stress ratio of 0.1. Here, The Paris–Erdogan law was adopted to describe the relationship between *da*/*dN* and Δ*K*. The Paris equations for the BM, HAZ and WM specimens were determined based on fatigue crack growth data. As shown in Figure 6, there is a linear relation between *da*/*dN* and Δ*K* in log-log coordinate, which can be expressed in Equations (5)–(7), respectively.

For the BM specimen

(5) $$\frac{da}{dN}=3.29\times 10^{-8}{\left(\Delta K_{I}\right)}^{2.81}$$

For the HAZ specimen

(6) $$\frac{da}{dN}=6.37\times 10^{-8}{\left(\Delta K_{I}\right)}^{3.69}$$

And, for the WM specimen

(7) $$\frac{da}{dN}=7.87\times 10^{-8}{\left(\Delta K_{I}\right)}^{3.84}$$

As shown in Equations (5)–(7), the lowest exponent m value of 2.81 was obtained for the BM specimen, followed by the value of 3.69 for the HAZ specimen and 3.84 for the WM specimen, depicting a relatively significant difference in the slope of the Paris curve for each type of specimen. It was generally recognized that lower *m* value can be regarded as smaller the change of crack propagation rate for a given stress intensity factor range [32]. Hence, it can be inferred that the BM had a lower variation of the FCG rate than the HAZ and the WM, with regard to the slope of the Paris curve. Also, similar results were previously reported by Xie [33] for the 316 steel and by Belattar [34] for 304 L steel. It should also be noted that for the same value of Δ*K*, the WM specimen tended to have the highest FCG rate, which resulted in reducing fatigue life of weld joints. Generally, the inhomogeneous distributions of microstructure and the presence of residual stresses could lead to differences in FCG rates of weld joints. In order to further investigate potential reasons for differences in FCG rate for the BM, HAZ and WM specimens, the metallographic structure, fractograph and residual stress distribution were compared and analyzed in the next several sections.

### 3.3. Metallographic Microstructure

**Figure 7:**
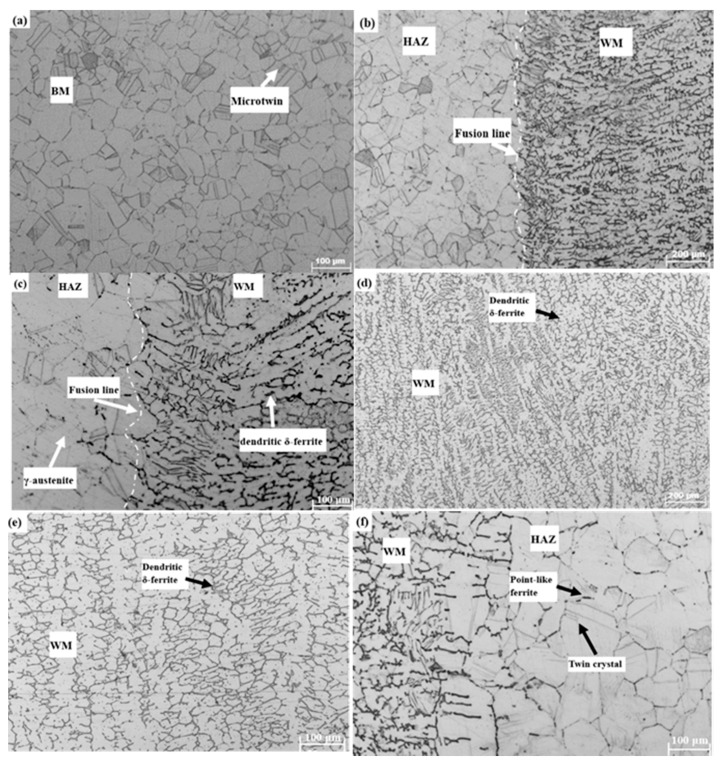
Metallographic structures of weld joints (**a**) the BM; (**b**) the HAZ + BM (100×); (**c**) the HAZ + BM (200×); (**d**) the WM (100×); (**e**) the WM (200×); (**f**) the HAZ.

The metallographic microstructures of the BM, the HAZ and the WM specimens under different magnifications are depicted in Figure 7.

#### 3.3.1. Metallographic Microstructure of the BM

It was observed that the BM was composed of a single phase of γ-austenite with an average grain size of 45 μm, as shown in Figure 7a. The grain size of γ-austenite was relatively uniform and the grain boundaries of austenite phase were clear. Besides, there were a few microtwins in the γ-austenite grains. 

#### 3.3.2. Metallographic Microstructure of the Intermediate Zone

Figure 7b,c show the intermediate zone between the HAZ and WM at 200 times and 400 times magnifications, respectively. Apparently, metallographic microstructures of the HAZ (on the left) were completely different from those of the WM (on the right). The metallographic microstructures of the WM were shown in Figure 7d,e. The WM consisted of two primary phases: the γ-austenite matrix and the dendritic δ-ferrite. During the cooling process, δ-ferrite phase precipitated in the WM and then transformed into γ-austenite phase. The cooling speed was too fast that only a small portion of δ → γ transformation could be achieved. Some Cr-rich austenite nuclei were preserved and formed into wormlike or skeletal δ-ferrite [35]. Remarkably, metallographic microstructures in the edge of the WM were heterogeneous, which was potentially harmful to fatigue crack growth properties of the WM, as seen in Figure 7b,c. 

#### 3.3.3. Metallographic Microstructure of the HAZ

Moreover, the HAZ consisted of inhomogeneous blocky austenite and point-like ferrite, as shown in Figure 7f. When the alloy was heated to a high temperature of 1150 °C (γ + δ), γ → δ transformation occurred. Normally, δ-ferrite nucleus easily emerged at the place where Cr is concentrated. Most δ phases transformed into solid γ phases during the cooling process and only core parts of δ nucleus were retained [36]. The average size of austenite phase in the HAZ was approximately 65 μm, relatively larger than the grain size in the BM, consistent with experimental results in the previous studies [37,38]. There has been a theoretical dispute concerning the effect of grain size on the FCG rates of metal alloys. Some researchers thought that small grains created more grain boundaries, which could retard the motion of dislocation and thus decrease the FCG rates [39]. However, many scholars [40,41] proposed that coarse grain tended to introduce slip planarity and crack closure phenomena, which decreased the FCG rate. In this paper, it was observed that the increase of grain size showed negative effects on the FCG rate. 

Through comprehensive comparison and overall analysis, it was concluded that the size and morphology of metallographic microstructures in the WM, the HAZ and the BM were significantly different, which could partially explain the potential reason for differences in FCG rate for the BM, HAZ and WM specimens.

### 3.4. Fracture Morphologies of the BM, the HAZ and the WM

**Figure 8:**
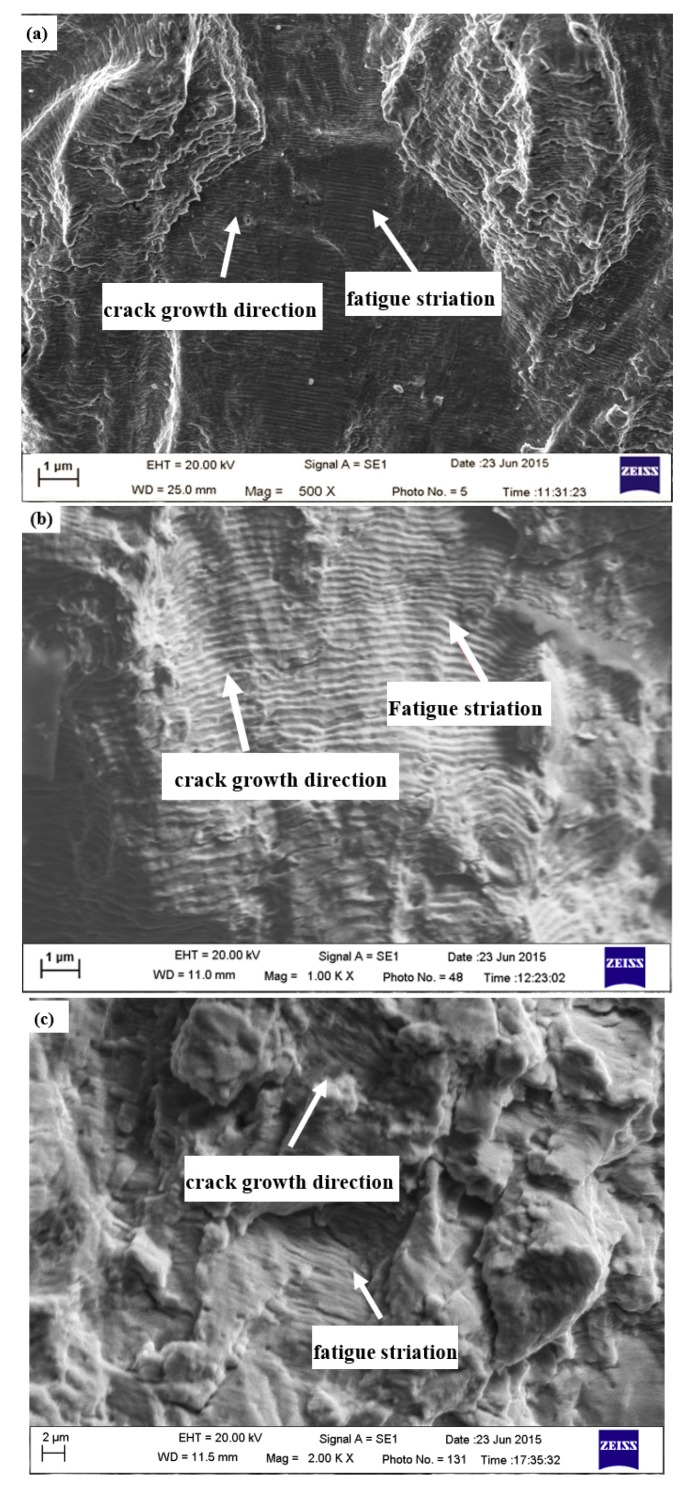
Fracture surface in FCG area: (**a**) the BM; (**b**) the HAZ specimen; and (**c**) the WM specimen.

**Figure 9:**
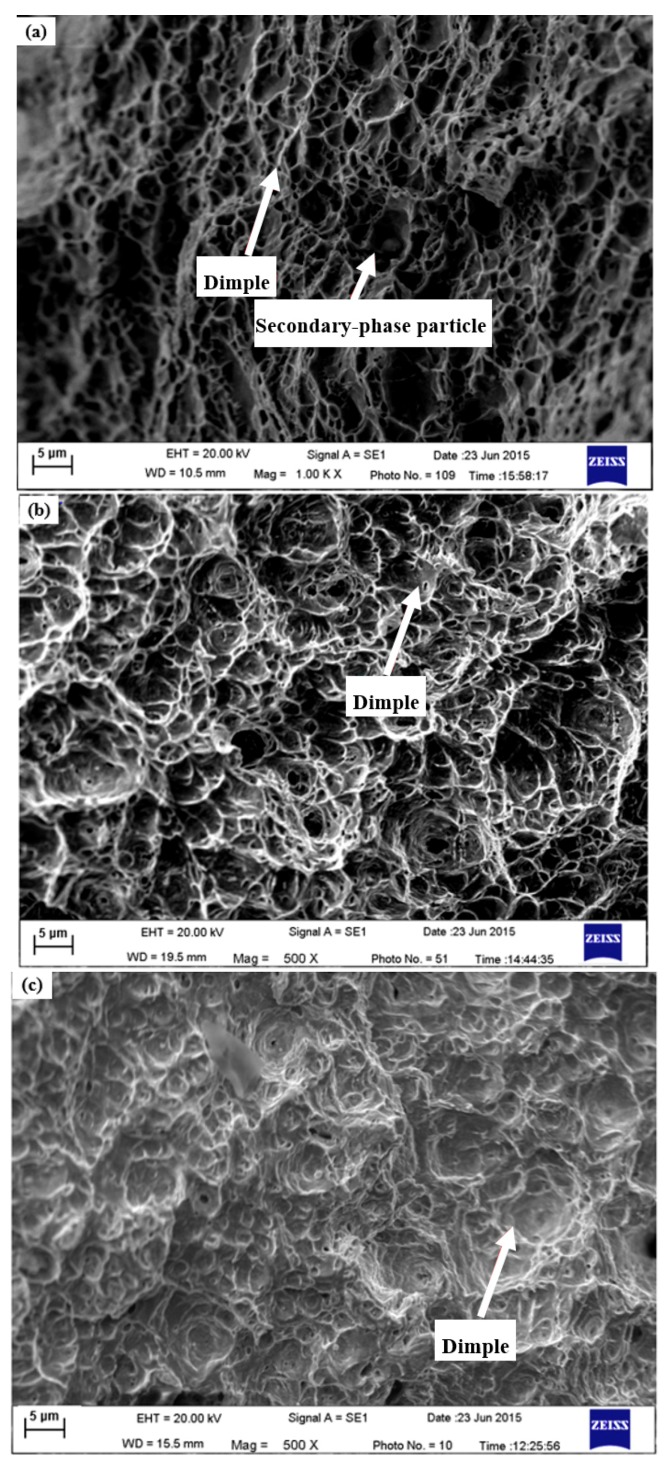
Fracture surface in ultimate fracture area: (**a**) the BM; (**b**) the HAZ specimen; and (**c**) the WM specimen.

Fracture morphologies of the BM, the HAZ and the WM were investigated to explain the difference in FCG rate, as shown in Figure 8 and Figure 9. Typically, fatigue fracture morphology in materials under cyclic loading consists of two typical regions, namely, the crack growth and the ultimate fracture regions [42]. In general, the surface micromorphology in two areas varies considerably.

#### 3.4.1. FCG Region

Figure 8 shows the crack growth region in the BM, the HAZ and the WM specimens at a crack length of approximately 2.7 mm. The fatigue striations, which were perpendicular to the crack growth path, could be observed in all types of specimens. It was generally known that the fatigue striation is a typical feature on the fracture surface of ductile metals and alloys. In the BM and HAZ specimen, the crack growth region demonstrated a relatively flattened surface where fatigue striations could be easily found, as shown in Figure 8a,b. The average values of striation spacing were 0.07 μm for the BM and 0.11 μm for the HAZ. However, the FCG region in the WM specimen showed a relatively rugged surface. It seemed that fatigue striations in the WM were in an intermittent state and hard to be observed. Compared to the BM and the HAZ, the WM specimen had a relatively higher value of striation spacing of around 0.13 μm. The observation results of the fatigue striation spacing were similar with the results of earlier works by Xie and Fan [33,43]. Normally, the fatigue striation spacing was roughly equivalent to the FCG whereby each fatigue striation corresponded to one loading cycle. It can be inferred that the WM specimen had the largest FCG, followed by the HAZ specimen, and the BM had the lowest FCG. Experimental results of the fatigue striation were basically consistent with the FCG results provided by Figure 6.

#### 3.4.2. Ultimate Fracture Region

The fracture surfaces of ultimate fracture region in the BM, the HAZ and the WM specimens are presented in Figure 9. It was observed that the fracture surfaces in all three types of the specimens existed equiaxial dimples with particles of the secondary phases, indicating a ductile fracture mode in the ultimate fracture area, as seen in Figure 9. The mechanisms of the dimple can be ascribed to the nucleation, growth and pro-life of micro-voids formed by inclusions or secondary phase particles. In general, under the same fatigue fracture condition, the plasticity of a material rises with increasing of the depth and decreasing of the size of the dimples [44]. As seen in Figure 9c, the depth of dimple in the WM was shallower than that in the BM and the HAZ, implying the larger plasticity of the material in comparison to the BM and HAZ.

### 3.5. Residual Stresses of the Welded Joint

**Figure 10:**
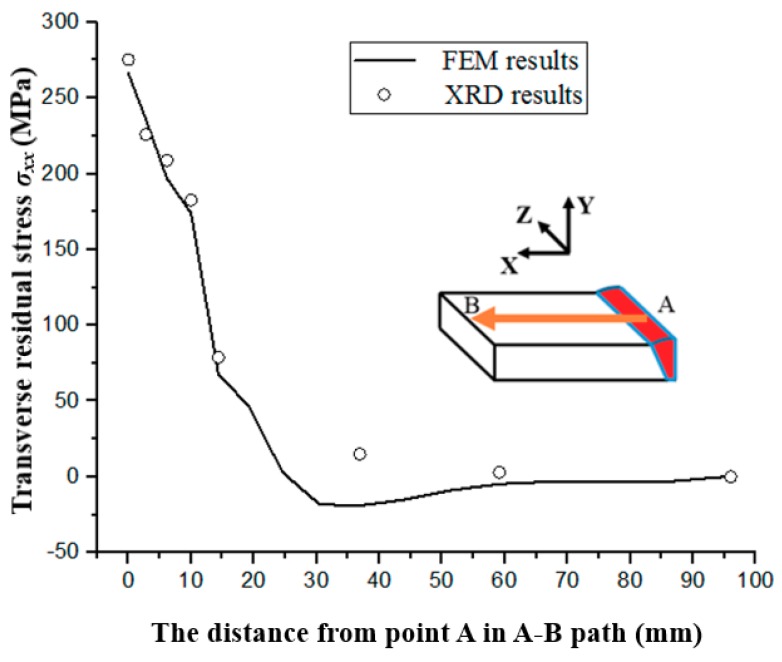
Transverse residual stress *σ_xx_* along A–B path.

The FE simulations and the XRD experiments were performed to evaluate residual stress distribution of the BM, the HAZ and the WM of weld specimens. Figure 10 shows both predicted and experimental residual stresses along the A–B path. Specially, the hollow dots shown in Figure 10 represented the experimental data obtained by the XRD analysis. The *σ_xx_* corresponded to the transverse residual stress in the same direction of the fatigue loading. It could be found that the experimental residual stress results were in good agreement with the simulated ones. For the WM and the HAZ, the *σ_xx_* was in a high tension stress state. The peak value of the *σ_xx_* of 275 MPa occurred in the center of the WM, then decreased sharply with the distance from the point A. The *σ_xx_* dropped to an average value of 200 MPa in the HAZ and fluctuated at zero in the BM.

These obtained results for the residual stress distribution were similar to the previous study findings [12]. In the absence of residual stress, the stress ratio *R* could be calculated by dividing the minimum stress $\sigma_{\min}$ by the maximum stress $\sigma_{\max}$, as shown in Equation (8)

(8) $$R=\frac{\sigma_{\min}}{\sigma_{\max}}$$

**Figure 11:**
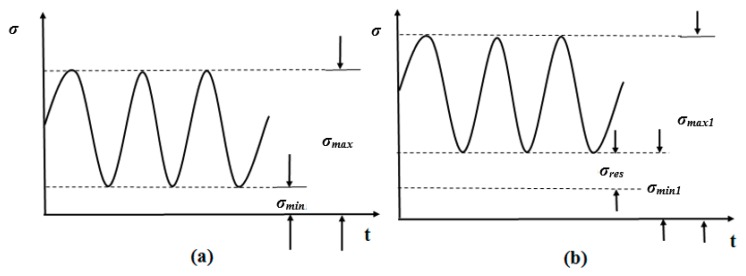
The fatigue cyclic stress: (**a**) without $\sigma_{res}$; (**b**) with $\sigma_{res}$.

And, considering the residual stress presence, the new stress ratio $R_{1}$ could be evaluated as shown in Figure 11.

(9) $$R_{1}=\frac{\sigma_{\min1}}{\sigma_{\max1}}=\frac{\sigma_{\min}+\sigma_{res}}{\sigma_{\max}+\sigma_{res}}$$

By substituting $\sigma_{\min}=R\sigma_{\max}$ from Equation (8):

(10) $$R_{1}=\frac{\left(R\sigma_{\max}+R\sigma_{res})+(\sigma_{res}-R\sigma_{res}\right)}{\sigma_{\max}+\sigma_{res}}=R+\frac{\sigma_{res}(1-R)}{\sigma_{\max}+\sigma_{res}}$$

Considering the positive *R* ratio adopted in this paper:

(11) 0<R<1

When the $\sigma_{res}$ was in the state of tensile stress, it was considered as numerically positive

(12) $$\sigma_{res}>0$$

Substituting Equations (11) and (12) into Equation (9), we obtained

(13) $$R_{1}>R$$

According to the Equation (13), tensile residual stresses in the WM and the HAZ increased the stress ratio *R*. It was well documented that the FCG rate *da*/*dN* is a function of the stress ratio *R*, the higher *R*, the higher *da*/*dN* [45]. Thus, it could be inferred that the tensile residual stress was one of the possible reasons why the WM and the HAZ had high FCG rates.

## 4. Conclusions

In this paper, the fatigue crack propagation behavior of the WM, the HAZ and the BM in 06Cr19Ni10 stainless steel butt joints was investigated and analyzed. Furthermore, metallographic structures, fracture morphology and residual stress of the specimens were also studied and compared to describe the difference in FCG rates among the three types of specimens. The main conclusions and important outcomes of the present research are summarized below:

1. In the BM, HAZ and WM specimens, the original mixed-mode cracks changed into the mode I propagating crack. The specimen’s type and the crack location had a negligible effect on the FCG path in the weld joints.
2. FCG test results indicated that the WM specimen had the largest FCG rate, followed by the HAZ specimen. The BM specimen had the lowest FCG rate.
3. Metallographic analysis showed that the size and morphology of metallographic microstructures in the WM, the HAZ and the BM were significantly different, which could partially explain the reason for difference in FCG rates for the BM, HAZ and WM specimens.
4. According to the fracture morphology, in the FCG area, the WM specimen had the largest striation spacing, followed by the HAZ specimen, and the BM had the lowest striation spacing. In the ultimate fracture area, depth of the dimple in the WM was shallower than that in the BM and the HAZ, implying larger plasticity of the material.
5. The FE simulation and the XRD experiments showed that the high values of tensile residual stresses were found in the WM and the HAZ, which could be accounted for higher FCG rates of the WM and HAZ.
6. The differences in fatigue crack growth behavior of the BM, WM, HAZ had been assumed to stem from the combination of microstructure difference and tensile residual stresses. However, the tensile residual stresses were considered to induce a more significant impact on the FCG rates of the welded joint.

## Figures and Tables

**Table 1:** Chemical component of the BM and filler (wt %).

	Cr	Ni	C	Si	Mn	S	Fe
BM (06Cr19Ni10)	17.2	8.10	0.043	0.46	1.12	0.001	Remainder
Filler (ER308L)	20.1	10.2	0.028	0.61	1.85	0.008	Remainder

**Table 2:** Technical parameters of the MIG welding.

Parameter	Welding Voltage (V)	Welding Current (A)	Welding Speed (cm/min)	Filler Material	Shielding Gas Type
Value	24.5	217	40	ER308L	Argon, O_2_

## Nomenclature

*a*	crack length (mm)
*C*	Paris material constant
BM	base metal
*da*/*dN*	crack growth rate (mm/cycle)
$d_{0}$	crystallographic d-spacing under stress-free condition (μm)
*d_ψ_*	crystallographic d-spacing under stress condition (μm)
*E*	the Young’s modulus (GPa)
FCG	fatigue crack growth
FEA	finite element analysis
*h*	crystal face exponent
HAZ	heat affected zone
*k*	crystal face exponent
*l*	crystal face exponent
*m*	Paris exponent
*R*	stress ratio
*U*	welding voltage (V)
*υ*	Poisson’s ratio
*I*	welding current (A)
XRD	X-Ray Diffraction
β	inclined angle of pre-crack (°)
*σ_xx_*	transverse residual stress (MPa)
*σ_eff_*	effective stress (MPa)
σ_ys_	yield strength (MPa)
*σ_res_*	residual stress (MPa) *σ_xx_*
ψ	the incident angle (°)
Δ*K*	stress intensity factor (SIF) range
Δ*K_I_*	stress intensity factor range of mode I
